# Chrestenson transform FPGA embedded factorizations

**DOI:** 10.1186/s40064-016-3162-9

**Published:** 2016-09-08

**Authors:** Michael J. Corinthios

**Affiliations:** Ecole Polytechnique de Montréal, Campus Université de Montréal, 2500 Chemin de Polytechnique, Montréal, QC H3T 1J4 Canada

**Keywords:** Spectral analysis, Generalised spectral analysis, Generalised Walsh transform, Discrete Chrestenson transform, Discrete Fourier transform, Parallel processing, Hypercube transformations, General-radix matrix factorization

## Abstract

Chrestenson generalized Walsh transform factorizations for parallel processing imbedded implementations on field programmable gate arrays are presented. This general base transform, sometimes referred to as the Discrete Chrestenson transform, has received special attention in recent years. In fact, the Discrete Fourier transform and Walsh–Hadamard transform are but special cases of the Chrestenson generalized Walsh transform. Rotations of a base-*p* hypercube, where *p* is an arbitrary integer, are shown to produce dynamic contention-free memory allocation, in processor architecture. The approach is illustrated by factorizations involving the processing of matrices of the transform which are function of four variables. Parallel operations are implemented matrix multiplications. Each matrix, of dimension *N* × *N*, where *N* = *p*^*n*^, *n* integer, has a structure that depends on a variable parameter *k* that denotes the iteration number in the factorization process. The level of parallelism, in the form of *M* = *p*^*m*^ processors can be chosen arbitrarily by varying *m* between zero to its maximum value of *n* − 1. The result is an equation describing the generalised parallelism factorization as a function of the four variables *n*, *p*, *k* and *m*. Applications of the approach are shown in relation to configuring field programmable gate arrays for digital signal processing applications.

## Background

Applications of the Discrete Fourier, Walsh–Hadamard and Chrestenson generalized Walsh CGW transforms in spectral analysis and digital signal processing (Corinthios 1985, 2009; Bespalov 2010) have received particular attention in recent years thanks to rapid advances of microelectronics in general and field programmable gate arrays FPGAs in particular. The search for higher processing speeds through increasing levels of parallelism motivate the search for optimal transform factorizations.

In this paper a formalism and an algorithm for configuring and sequencing parallel processors implementing factorizations of the (‘Discrete’) Chrestenson generalized Walsh CGW transform are presented. This general base transform has received special attention in recent years. In fact, Discrete Fourier transform and Walsh–Hadamard transform are but special cases of the CGW transform. The architecture of a digital signal processor is defined as optimal if it leads to a minimization of addressing requirements, of shuffle operations and of the number of required memory partitions (Corinthios 1994). The factorizations are developed with a view to implementation as embedded architectures of presently available FPGAs (Harmut et al. 2010; Huda et al. 2014).

The algorithms and corresponding architectures relate to general base matrix factorizations (Corinthios 2009). Rotations of a base-*p* hypercube, where *p* is an arbitrary integer, produce dynamic memory allocation, in processor architecture. The approach produces factorizations involving the processing of matrices of the transform which are function of four variables. Parallel operations are implemented matrix multiplications. Each matrix, of dimension *N* × *N*, where *N* = *p*^*n*^, *n* integer, has a structure that depends on a variable parameter *k*. The level of parallelism, in the form of *M* = *p*^*m*^ processors can be chosen arbitrarily by varying *m* between zero to its maximum value of *n* − 1. The result is an equation describing the generalised parallelism factorization as a function of the four variables *n*, *p*, *k* and *m.* Applications of the approach are shown in relation to configuring field programmable gate arrays for digital signal processing applications.

Hypercube transformations have been applied to diversified problems of information processing. The present paper describes an approach for FPGA parallel processor configuration using an *arbitrary number M* of general-base processing elements, where *M* = *p*^*m*^, *p* being the general radix (base) of factorization. The input data vector dimension *N*, or input data matrix dimension *N* × *N*, where *N* = *p*^*n*^, the radix, or base, *p* of factorization of the transformation matrix, the number of processors *M*, and the span of the matrix, that is, the spacing between data simultaneously accessed are all variable. A unique optimal solution yielding a progressive degree parallel to massively parallel optimal architectures is presented.

## Matrix structures

In what follows some definitions relating to the special structure of sparse, permutation and transformation matrices (Corinthios 1994) are employed. In particular matrix span is taken to mean the distance between two successive nonzero elements along a row or a column. A fixed topology processor is one that accesses data in a fixed geometry pattern where data points are equidistant throughout the different iterations, thus requiring no addressing. A shuffle-free algorithm is one that necessitates no data shuffling between iterations. A *p*^*k*^-optimal algorithm is one that requires access of matrix elements which are spaced by a minimum distance of *N*/*p*^*k*^ elements. In addition we adopt the following definitions.

### General base processing element

In what follows a general-base processing element PE with a base, or radix, *p* is a processor that receives simultaneously *p* input operands and produces simultaneously *p* output operands. The PE in general applies arithmetic or weighting operations on the input vector to produce the output vector. In matrix multiplication operations for example the PE applies a *p* × *p* matrix to the *p*-element input vector to produce the *p*-element output vector. The matrix elements may be real or complex.

Due to the diversified general applicability of such a processing element a universal processing element (UPE), which can be constructed in a 3D-type architecture has been proposed (Corinthios 1985). In the present context a UPE may be seen simply as a general base-*p* processing element PE as defined above, accepting *p* inputs, weighting them by the appropriate *p* × *p* matrix and producing *p* output operands.

### Pilot elements, pilots matrix

Similarly to signals and images an *N* × *N* matrix may be sampled and the result is “impulses”, that is, isolated elements in the resulting *N* × *N* samples matrix. We shall assume uniform sampling of rows and columns yielding *p* uniformly spaced samples from each of *p* rows and element alignment along columns, that is, *p* uniformly spaced samples along columns as well as rows. The samples matrix which we may refer to as a “frame” thus contains *p* rows of *p* equally spaced elements each, a rectangular grid of *p*^2^ impulses, which we may refer to as “poles”, which we shall call a “dispatch”. With *N* = *p*^*n*^ the *N*^2^ elements of the “main” (or “parent”) matrix, that is, the original matrix before sampling, may be thus decomposed into *N*^2^/*p*^2^ = *p*^*n*−2^ such dispatches.

By fixing the row sampling period as well as the column sampling period, the row and column spans of the resulting matrix are known. It therefore suffices to know the coordinates (indices) of the top left element, that is, the element with the smallest of indices, of a dispatch to directly deduce the positions of all its other poles. The top left element acts thus as a reference point, and we shall call it the “pilot element”. The other *p*^2^ − 1 elements associated with it may be called its “satellites”.

In other words if the element *a*_*ij*_ of *A* is a pilot element, the dispatch consists of the elements

$$ a_{i + kc,j + lr} ;\quad k = 0,1, \ldots ,\,p - 1,\quad l = 0,\,1, \ldots ,p - 1 $$

*c* and *r* being the column and row element spacing (spans), respectively.

A processing element assigned to a pilot element can thus access all *p*^2^ operands of the dispatch, having deduced their positions knowing the given row and column spans.

Since each pilot element of a frame originated from the same position in the parent matrix we can construct a “pilots matrix” by keeping only the pilot elements and forcing to zero all other elements of the parent matrix. The problem then is one of assignment, simultaneous and/or sequential, of the *M* = *p*^*m*^ processors to the different elements of the pilots matrix.

### Hypercube dimension reduction

The extraction of a pilots matrix from its parent matrix leads to a dimension reduction of the hypercube representing its elements. The dimension reduction is in the form of a suppression, that is, a forcing to zero, of one of the hypercube digits. Let *C* = (*j*_*n*−1_, …, *j*_1_*j*_0_), *j*_*k*_ ∊ {0, 1, 2, …, *p* − 1} be an *n*-digit base-*p* hypercube. We will write $C_{{\bar{k}}}$ to designate the hypercube *C* with the digit *k* suppressed, that is, forced to zero. Several digits can be similarly suppressed. For example, $C_{{\overline{2} ,\overline{4} }} = \left( {j_{n - 1} \ldots j_{5} 0j_{3} 0j_{1} j_{0} } \right)$, and $C_{{\overline{n - 1} }} = \left( {0j_{n - 2} \ldots j_{1} j_{0} } \right)$.

## Parallel configuration algorithm

A sequence of perfect shuffle operations effected through simple hypercube transformations can be made to broadcast the parallel configuration and access assignments to the different processors. The overall approach is described by the following algorithm.
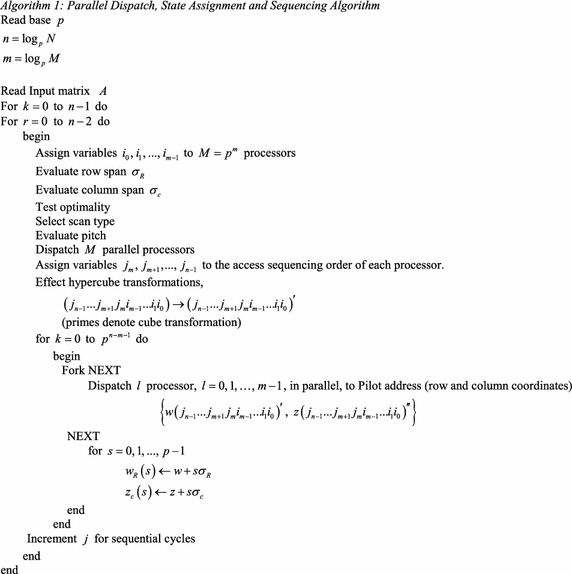


The parallel dispatch, state assignment and sequencing Algorithm 1 dispatches the *M* = *p*^*m*^ processors for each stage of the matrix factorization. The base-*p m*-tuple (*i*_*m*−1_*i*_*m*−2_…*i*_1_*i*_0_) is assigned to the parallel processors. The (*n* − *m*) tuple (*j*_*n*−1_*j*_*n*−2_…*j*_*m*_) is assigned to the sequencing cycles of each processor. The algorithm subsequently applies hypercube transformations as dictated by the type of matrix, the stage of matrix factorization and the number of dispatched processors. It tests optimality to determine the type of scan of matrix elements to be applied and evaluates parameters such as pitch and memory optimal queue length, to be defined subsequently, it accesses the pilot elements and their satellites, proceeding to the parallel dispatch and sequencing of the processing elements.

Each processing element at each step of the algorithm thus accesses from memory its *p* input operands and writes into memory those of its output operands. The algorithm, while providing an arbitrary, generalised, level of parallelism up to the ultimate massive parallelism, produces optimal multiprocessing machine architecture minimizing addressing, the number of memory partitions as well as the number of required shuffles. Meanwhile it produces virtually wired-in pipelined architecture and properly ordered output.

## General matrix decomposition

In developing techniques for the general-base factorization of transformation matrix multiplications it is convenient to effect a decomposition of a matrix into the sum of matrices. To this end let us define an “impulse matrix” as the matrix *δ*(*i*, *j*) of which all the elements are zero except for the element at position (*i*, *j*), that is,

1 $$ \left| {\delta \left( {i,j} \right)} \right|_{uv} = \left\{ \begin{array}{ll} 1,&\quad u = i,\,v = j \hfill \\ 0,&\quad otherwise \hfill \\ \end{array} \right.. $$

An *N* × *N* matrix *A* having elements [*A*]_*i*,*j*_ = *a*_*ij*_ can be written as the sum

2 $$ \begin{aligned} A & = a_{0,0} \delta \left( {0,0} \right) + a_{0,1} \delta \left( {0,1} \right) + a_{0,2} \delta \left( {0,2} \right) + \cdots + a_{1,0} \delta \left( {1,0} \right) \\ & \quad + \,a_{1,1} \delta \left( {1,1} \right) + \cdots + a_{N - 1,N - 1} \delta \left( {N - 1,N - 1} \right) \\ \end{aligned} $$

where the *δ*(*i*, *j*) matrices are of dimension *N* × *N* each. The matrix *A* can thus be written in the form

3 $$ A = \sum\limits_{i = 0}^{N - 1} {\sum\limits_{j = 0}^{N - 1} {a_{i,j} } } \delta \left( {i,j} \right). $$

Furthermore, in the parallel processing of matrix multiplication to a general base *p* it is convenient to decompose an *N* × *N* matrix with *N* = *p*^*n*^ as the sum of dispatches, a dispatch being, as mentioned earlier, a matrix of *p*^2^ elements arranged in a generally rectangular *p* × *p* pattern of *p* columns and *p* rows. Denoting by *σ*_*R*_ and *σ*_*C*_ the row and columns spans of a dispatch we can decompose a matrix *A* into the form

4 $$ A = \sum\limits_{i = 0}^{N/p - 1} {\sum\limits_{j = 0}^{N/p - 1} {\sum\limits_{k = 0}^{p - 1} {\sum\limits_{l = 0}^{p - 1} {a_{{i + k\sigma_{C} ,j + l\sigma_{R} }} } } } } \delta \left( {i + k\sigma_{C} ,j + l\sigma_{R} } \right). $$

More generally we may wish to decompose *A* in an order different from the uniform row and column scanning as in this last equation. In other words we may wish to pick the dispatches at an arbitrary order rather than in sequence. As mentioned above, we shall call the top left element the pilot element and its *p*^2^ − 1 companions its satellites. In this last equation the pilot elements are those where *k* = 1 = 0.

To effect a parallel matrix decomposition to a general base-*p* we use hypercubes described by base-*p* digits. The order of accessing the different dispatches is made in relation to a main clock. The clock *K* is represented by the hypercube to base *p* as

5 $$ K \simeq \left( {k_{n - 1} \ldots k_{1} k_{0} } \right)_{p} ;\quad k_{i} \in \left\{ {0,1, \ldots ,p - 1} \right\} $$

Its value at any time is given by

6 $$ K = \sum\limits_{t = 0}^{n - 1} {p^{t} } k_{t} . $$

At each clock value *K* a set of M UPE’s (PE’s) is assigned a set of *M* dispatches simultaneously. We will reserve the symbols *w* and *z* to designate the row and column indices of a pilot element at clock *K*. In other words, at clock *K* each selected pilot element shall be designated *a*_*w*,*z*_, that is, [*A*]_*w*,*z*_ where *w* and *z* are functions of *K* to be defined. They will be determined in a way that optimizes the parallel and sequential operations for the given matrix structure and the number *M* = *p*^*m*^ of available UPE’s.

With *M* = *p*^*m*^ base-*p* processing elements the hypercube representing *K* shall be re-written in the form

7 $$ K \simeq \left( {j_{n - 1} \ldots j_{m + 1} j_{m} i_{m - 1} \ldots i_{1} i_{0} } \right)_{p} $$

where we have written

8 $$ k_{t} = \left\{ {\begin{array}{*{20}l} {i_{t} ,} \hfill & {\quad t = 0,1, \ldots ,m - 1} \hfill \\ {j_{t} ,} \hfill & {\quad t = m,m + 1, \ldots ,n - 1.} \hfill \\ \end{array} } \right. $$

The *m*-sub-cube (*i*_*m*−1_, …, *i*_1_, *i*_0_) designates operations performed in parallel. The remaining (*n* − *m*)-sub-cube (*j*_*n*−1_, …, *j*_*m*+1_, *j*_*m*_) designates operations performed sequentially by each of the *M* dispatched parallel processors. With *M* = *p*^*m*^ processors dispatched in parallel at clock *K* ≃ (*j*_*n*−1_…*j*_*m*+1_*j*_*m*_*i*_*m*−1_…*i*_1_*i*_0_)_*p*_ the matrix *A* can be decomposed in the form

9 $$ \begin{aligned} A & = \sum\limits_{{k_{n - 2} = 0}}^{p - 1} \ldots \sum\limits_{{k_{m + 1} = 0}}^{p - 1} {\sum\limits_{{k_{m} = 0}}^{p - 1} {} } \\ & \quad \left\langle {\sum\limits_{{k_{m - 1} = 0}}^{p - 1} \ldots \sum\limits_{{k_{1} = 0}}^{p - 1} {\,\sum\limits_{{k_{0} = 0}}^{p - 1} {\,\sum\limits_{l = 0}^{p - 1} {\,\sum\limits_{k = 0}^{p - 1} {a_{{w\left( {k_{0} ,k_{1} , \ldots ,k_{n - 1} } \right) + k\sigma_{C} ,z\left( {k_{0} ,k_{1} , \ldots ,k_{n - 1} } \right) + l\sigma_{R} }} } } } } } \right. \\ & \quad \left. {\delta \left[ {\left\{ {w\left( {k_{0} ,k_{1} , \ldots ,k_{n - 2} } \right) + k\sigma_{C} } \right\},\left\{ {z\left( {k_{0} ,k_{1} , \ldots ,k_{n - 2} } \right) + l\sigma_{R} } \right\}} \right]} \right\rangle \\ \end{aligned} $$

where the “parentheses” 〈 and 〉 enclose the elements accessed in parallel. In what follows we write *P*_*ν*,*μ*_ to designate the pilot element of processor no. *ν* at real time clock *μ*.

## Application to the CGW transforms

The lowest order base-*p* Chrestenson generalised Walsh CGW “core matrix” is the *p*-point the Discrete Fourier matrix

10 $$ W_{p} = \frac{1}{\sqrt p }\left[ {\begin{array}{*{20}c} {w^{0} } & {w^{0} } & \cdots & {w^{0} } \\ {w^{0} } & {w^{1} } & \cdots & {w^{p - 1} } \\ \vdots & {} & {} & {} \\ {w^{0} } & {w^{p - 1} } & \cdots & {w^{{\left( {p - 1} \right)^{2} }} } \\ \end{array} } \right] $$

where

11 $$ w = \exp \left( { - j2\pi /p} \right),\quad j = \sqrt { - 1} . $$

In the following, for simplicity, the scaling factor $1/\sqrt p$ will be dropped. We start by deriving three basic forms of the Chrestenson (generalised Walsh GW) transform in its three different orderings: in natural order CGWN, in Walsh–Paley order CGWP and in Walsh–Kaczmarz order CGWK.

### The CGWN transformation matrix

The CGWN transformation matrix *W*_*N*_ for *N* = *p*^*n*^ data points is obtained from the generalised-Walsh core matrix *W*_*p*_ by the Kroneker multiplication of *W*_*p*_ by itself *n* times.

12 $$ W_{N,nat} = W_{p} \times W_{p} \times \cdots \times W_{p} \left( {n\,\text{times}} \right) = W_{p}^{\left[ n \right]} . $$

### CGWP transformation matrix

The generalised Walsh transform in the CGWP order is related to the transform in natural order by a digit-reverse ordering. The general-base digit reverse ordering matrix *K*_*N*_^(*p*)^ can be factored using the general-base perfect shuffle permutation matrix *P*^(*p*)^, also denoted simply *P*, and Kroneker products

13 $$ K_{N}^{\left( p \right)} = \prod\limits_{i = 0}^{n - 1} {\left( {P_{{p^{{\left( {n - i} \right)}} }}^{\left( p \right)} \times\,I_{{p^{i} }} } \right)} $$

where *I*_*K*_ is the identity matrix of dimension *K*.

Operating on a column vector *x* of dimension *K* the base-*p* perfect shuffle permutation matrix of dimension *K* × *K* produces the vector

14 $$ P_{K} x = \left[ {x_{0} ,x_{K/p} ,x_{2K/p} , \ldots ,x_{{\left( {p - 1} \right)K/p}} ,x_{1} ,x_{K/p + 1} , \ldots ,x_{2} ,x_{K/p + 2} , \ldots ,x_{K - 1} } \right] $$

The CGWP matrix *W*_*N*,*WP*_ can thus be written in the form

15 $$ W_{N,WP} = K_{N}^{\left( p \right)} W_{N,nat} = \prod\limits_{i = 0}^{n - 1} {\left( {P_{{p^{{\left( {n - 1} \right)}} }}^{\left( p \right)} \times\,I_{{p^{i} }} } \right)} \,W_{p}^{\left[ n \right]} . $$

### CGWK transformation matrix

The CGWK transformation matrix is related to the CGWP matrix through a *p*-ary to Gray transformation matrix *G*_*N*_^(*p*)^.

16 $$ W_{N,WK} = G_{N}^{(p)} W_{N,WP} . $$

The following factorizations lead to shuffle-free optimal parallel-pipelined processors.

### CGWN optimal factorization

A fixed topology factorization of the CGWN transformation matrix has the form

17 $$ W_{N,nat} = \prod\limits_{i = 0}^{n - 1} {P_{N} } C_{N} = \prod\limits_{i = 0}^{n - 1} {P_{N} } \left( {I_{N/p} \times W_{p} } \right) $$

which can be re-written in the form

18 $$ W_{N,nat} = P\left\{ {\prod\limits_{n = 0}^{n - 1} {CP} } \right\}P^{ - 1} = P\left\{ {\prod\limits_{n = 0}^{n - 1} F } \right\}P^{ - 1} $$

19 $$ C = C_{N} = I_{{p^{n - 1} }} xW_{p} $$

and *F* = CP, noting that the matrix *F* is *p*^2^-optimal.

### CGWP optimal factorization

We fixed topology factorization of the CGWP matrix has the form

20 $$ W_{N,WP} = \prod\limits_{i = 0}^{n - 1} {J_{i} } C_{N} $$

21 $$ J_{i} = \left( {I_{{P^{n - i - 1} }} \times\,P_{{p^{i + 1} }} } \right) = H_{n - i - 1} $$

Letting

$$ Q_{i} = C_{N} J_{i + 1} = C_{N} H_{n - i - 2} ,\quad i = 0,1, \ldots ,n - 2 $$

22 $$ Q_{n - 1} = C_{N} $$

we obtain

23 $$ W_{N,WP} = \prod\limits_{i = 0}^{n - 1} {Q_{i} } $$

where each matrix *Q*_*i*_, *i* = 0, 1, …, *n* − 2, is *p*^2^-optimal, while *Q*_*n*−1_ is *p*-optimal.

### CGWK optimal factorization

The fixed topology CGWK factorization has the form

24 $$ W_{N,WK} = P\left\{ {\prod\limits_{i = 0}^{n - 1} {P^{ - 1} } H_{i} C_{N} E_{i} } \right\}P^{ - 1} $$

Letting

25 $$ H_{i} = I_{{p^{i} }} \times P_{{p^{n - i} }} ,\quad E_{i} = I_{{p^{i} }} \times D_{{p^{n - i} }}^{\prime } $$

26 $$ D_{{p^{n} }}^{\prime } = \text{quasidiag}\left( {I_{{p^{n - 1} }} ,D_{{p^{n - 1} }} ,D_{{p^{n - 1} }}^{2} , \ldots ,D_{{p^{n - 1} }}^{{\left( {p - 1} \right)}} } \right) $$

A quasidiagonal matrix is a matrix containing matrices along its diagonal and null matrices elsewhere.

$$ D_{{p^{n - 1} }}^{i} = D_{p}^{i} \times I_{{p^{n - 2} }} $$

27 $$ D_{p} = \text{diag}\left( {w^{0} ,w^{ - 1} ,w^{ - 2} , \ldots ,w^{{ - \left( {p - 1} \right)}} } \right) $$

28 $$ W_{N,WK} = P\left\{ {\prod\limits_{i = 0}^{n - 1} {P^{ - 1} H_{i} G_{i} } } \right\}\,P^{ - 1} , $$

where

29 $$ G_{i} = C_{N} E_{i} $$

Letting

30 $$ S_{i} = P^{ - 1} H_{i} P = \left( {I_{{p^{i - 1} }} \times P_{{p^{n - i} }} \times I_{p} } \right) $$

we have

31 $$ W_{N,WK} = P^{2} \left\{ {\prod\limits_{i = 0}^{n - 1} {P^{ - 1} G_{i} S_{i + 1} } } \right\}\,P^{ - 1} $$

with

32 $$ S_{n - 1} = S_{n} = I_{N} $$

The factorization can also be re-written in the form

33 $$ W_{N,WK} = P\left\{ {\prod\limits_{i = 0}^{n - 1} {\Gamma_{i} } } \right\}\,P^{ - 1} , $$

where

34 $$ \begin{aligned}\Gamma _{i} & = P^{ - 1} G_{i} S_{i + 1} \\ & = P^{ - 1} G_{i} \left( {I_{{p^{i} }} \times P_{{p^{n - i - 1} }} \times I_{p} } \right)\,i = 1,2, \ldots ,n - 1; \\\Gamma _{0} & = G_{0} S_{1}. \\ \end{aligned} $$

The matrices Γ_*i*_ are *p*^2^-optimal, except for Γ_0_ which is maximal span. These are therefore optimal algorithms which can be implemented by an optimal parallel processor, recirculant or pipelined, with no shuffling cycle called for during any of the *n* iterations.

## Application to image processing

The potential in enhanced speed through high-level parallelism of the optimal algorithms is all the more evident within the context of real-time image processing applications. For 2D signals, algorithms of generalised spectral analysis can be applied on sub-images or on successive column-row vectors of the input image. Factorizations of the algorithms of the Chrestenson transform applied on an *N* × *N* points matrix *X* representing an image, with *N* = *p*^*n*^ can be written for the different transform matrices. The CGWN 2D transformation for optimal pipelined architecture can be written in the form

35 $$ \begin{aligned} Y_{nat} & = P\left\{ {\prod\limits_{i = 0}^{n - 1} F } \right\}\,P^{ - 1} \times \,\left[ {P\left\{ {\prod\limits_{i = 0}^{n - 1} F } \right\}\,P^{ - 1} } \right]^{T} \\ & = P\left\{ {\prod\limits_{i = 0}^{n - 1} F } \right\}\,P^{ - 1} \times P\left\{ {\prod\limits_{i = 0}^{n - 1} F } \right\}\,P^{ - 1} , \\ \end{aligned} $$

where *T* stands for transpose. The CGWP factorization can be written in the form

36 $$ \begin{aligned} Y_{WP} & = \prod\limits_{i = 0}^{n - 1} {Q_{i} \times \left( {\prod\limits_{i = 0}^{n - 1} {Q_{i} } } \right)^{T} } \\ & = \prod\limits_{i = 0}^{n - 1} {Q_{i} \times \prod\limits_{i = 0}^{n - 1} {Q_{n - i - 1}^{T} } } , \\ \end{aligned} $$

37 $$ Q_{i}^{T} = C_{N} \left( {I_{{p^{n - i - 1} }} \times P_{{p^{i + 1} }}^{ - 1} } \right) $$

The CGWK factorization for optimal pipelined architecture can be written in the form

38 $$ \begin{aligned} Y_{WK} & = P^{2} \left\{ {\prod\limits_{i = 0}^{n - 1} {\Gamma_{i} } } \right\}P \times \left[ {P^{2} \left\{ {\prod\limits_{i = 0}^{n - 1} {\Gamma_{i} } } \right\}P} \right]^{T} \\ & = P^{2} \left\{ {\prod\limits_{i = 0}^{n - 1} {\Gamma_{i} } } \right\}P \times P^{ - 1} \left\{ {\prod\limits_{i = 0}^{n - 1} {\Gamma_{n - i - 1}^{T} } } \right\}P^{ - 2} , \\ \end{aligned} $$

39 $$ \Gamma _{i}^{T} = \left( {I_{{p^{i} }} \times P_{{p^{n - i - 1} }}^{ - 1} \times I_{p} } \right)G_{i}^{ - 1} P. $$

These fast algorithms are all *p*^2^-optimal requiring no shuffling between iterations of a pipelined processor. In applying these factorizations the successive iterations are effected on successive sub-images such that after log_*p*_*N* stages the transform image *Y* is pipelined at the processor output. Applications include real-time processing of video signals.

The Discrete Fourier transform matrix for *N* points is the matrix *F*_*N*_ defined above in (10) with *p* replaced by *N* and the factor $1/\sqrt p$ optional:

40 $$ F_{N} = \left[ {\begin{array}{*{20}c} {w^{0} } & {w^{0} } & \cdots & {w^{0} } \\ {w^{0} } & {w^{1} } & \cdots & {w^{N - 1} } \\ {w^{0} } & {w^{2} } & \cdots & {w^{2(N - 1)} } \\ {w^{0} } & {w^{N - 1} } & \cdots & {w^{{(N - 1)^{2} }} } \\ \end{array} } \right]\, $$

For images the factorization leads to the optimal form

41 $$ Y_{F} = \left\{ {\prod\limits_{i = 0}^{n - 1} {F_{i} } } \right\} \times \left\{ {\prod\limits_{k = 0}^{n - 1} {F_{n - k - 1} } } \right\} $$

and for unidimensional signals the corresponding form for the Discrete Fourier matrix is

42 $$ F_{N} = \prod\limits_{i = 0}^{n - 1} {\left( {F_{i} } \right)} $$

43 $$ \begin{aligned} F_{i}&= U_{i} C_{i} \hfill \\ C_{i} &= C\,J_{i + 1} ,\quad i = 0,1, \ldots ,n - 1 \hfill \\ C_{n - 1} &= C \hfill \\ \end{aligned} $$

44 $$ \begin{aligned} U_{1} & = I_{N} \\ U_{i} & = I_{{p^{n - i - 1} }} \times D_{{p^{i + 1} }} = I_{{p^{n - i - 1} }} \times D_{{N/p^{n - i - 1} }} \\ D_{N/m} & = diag\left( {I_{N/(pm)} ,K_{m} ,K_{m}^{2} , \ldots ,K_{m}^{p - 1} } \right) \\ K_{t} & = diag\,\left( {w^{0} ,w^{t} , \ldots ,w^{{\left[ {N/(mp) - 1} \right]t}} } \right). \\ \end{aligned} $$

## Perfect shuffle hypercube transformations

The hypercube transformations approach is illustrated using the important matrices of the Chrestenson generalised Walsh–Paley (CGWP), generalised Walsh–Kaczmarz (CGWK) and the Discrete Fourier transforms.

We note that the matrices *C*_*k*_ in the Discrete Fourier transform expansion are closely related to the matrices *J*_*i*_ and *H*_*i*_ in the Chrestenson generalised Walsh Paley factorization. In fact the following relations are readily established:

45 $$ \begin{aligned} C_{N} &\,\triangleq\,C \\ C_{i} & = C\,J_{i + 1} = C\,H_{n - i - 2} = Q_{i} \\ \end{aligned} $$

where the equality ≜ sign means equal by definition.

46 $$ Q_{n - 1} = C_{n - 1} = C. $$

Therefore, the CGWP matrices *Q*_*i*_ are the same as the *C*_*i*_ matrices defined above and have the same structure as the *F*_*i*_ matrices in the Fourier matrix factorization. Writing

47 $$ B_{k} = CH_{k} $$

48 $$ H_{k} = I_{{p^{k} }} \,\times\,P_{{p^{n - k} }} $$

the post-multiplication by *H*_*k*_ has the effect of permuting the columns of *C* so that at row *w*,

49 $$ w\, \simeq \,\left( {0\,j_{n - 2} \, \ldots \,j_{1} \,j_{0} } \right) $$

the pilot element is at column *z* as determined by the permutation *H*_*k*_, that is,

50 $$ z\, \simeq \,\left( {j_{k} \,0\,j_{n - 2} \, \ldots \,j_{k + 1} \,j_{k - 1} \, \ldots \,j_{1} \,j_{0} } \right) $$

with the special case *k* = *n* − 2 producing

51 $$ z\, \simeq \,\left( {j_{n - 2} \,0\,j_{n - 3} \, \ldots \,j_{1} \,j_{0} } \right) $$

and that of *k* = *n* − 1 yielding

52 $$ z\, \simeq \,\left( {0\,j_{n - 2} \, \ldots \,j_{1} \,j_{0} } \right). $$

Alternatively, we can write *z* directly as a function of *w* by using previously developed expressions of permutation matrices. For example,

53 $$ B_{0} = CH_{0} = CP $$

and using the expression defining *P*, namely,

54 $$\begin{aligned} \left[ {P_{{p^{n} }}^{k} } \right]_{uv} &= \left\{ {\begin{array}{*{20}l} {1,} \hfill & {\quad u = 0,1, \ldots ,p^{n} - 1\,,} \hfill \\ {} \hfill & {\quad v = \left[ {u + \left( {u\,\bmod p^{k} } \right)\left( {p^{n} - 1} \right)} \right]/p^{k} } \hfill \\ {0,} \hfill & {\quad \text{otherwise}} \hfill \\ \end{array} } \right.\\ &\quad k = 0,1, \ldots ,N - 1 \end{aligned}$$

with *k* = 1, we can write

55 $$ z = \left[ {w + \left( {w\bmod p} \right)\,\left( {p^{n} - 1} \right)} \right]/p $$

a relation that defines the pilot elements matrix.

Similarly,

56 $$ B_{1} = C\,H_{1} = C\,\left( {I_{p} \times P_{{p^{n - 1} }} } \right) $$

and from the definition given in Corinthios (1994):

57 $$ \left[ {P_{i}^{t} } \right]_{uv} = \left\{ {\begin{array}{*{20}l} {1,} \hfill & {\quad u = 0,1, \ldots ,p^{n} - 1} \hfill \\ {} \hfill & {\quad v = p^{{i - t\bmod \left( {n - i} \right)}} [p^{ - i} \left( {u - u\bmod p^{i} } \right) + \{ [p^{ - i} (u - u\bmod p^{i} )]} \hfill \\ {} \hfill & {\quad \bmod p^{{t\bmod \left( {n - i} \right)}} \} \left( {p^{n - i} - 1} \right)] + u\bmod p^{i} } \hfill \\ {0,} \hfill & {\quad \text{otherwise}} \hfill \\ \end{array} } \right. $$

with *i* = 1 and *t* = 1 we have

58 $$ z = \left[ {p^{ - 1} \left( {w - w\,\bmod \,p} \right) + \left\{ {\left[ {p^{ - 1} \left( {w - w\,\bmod \,p} \right)} \right]\,\bmod \,p} \right\}\left( {p^{n - 1} - 1} \right)} \right] + w\,\bmod \,p. $$

Consider the permutation matrix

59 $$ R_{N} = R_{{p^{n} }} = I_{{p^{m} }} \times P_{{p^{j} }} \times I_{{p^{k} }} . $$

Let the base-*p* hypercube describing the order in a vector *x* of *N* = *p*^*n*^ elements be represented as the *n*-tuple.

60 $$ x \simeq \left( {j_{n - 1}   \ldots j_{1} j_{0} } \right)_{p} \quad j_{i} \in \left\{ {0, 1, \ldots , p - 1} \right\}. $$

The application of the matrix $R_{{p^{n} }}$ on the *n*-tuple vector *x*, results in the *n*-tuple:

61 $$ v = \left( {j_{n - 1}   \ldots j_{n - k + 1} \,j_{n - k} \,j_{m} \,j_{n - k - 1}   \ldots j_{m + 2} \,j_{m + 1} \,j_{m - 1}   \ldots j_{1} \,j_{0} } \right). $$

We note that with respect to *x* the left *k* digits and the right *m* digits are left unchanged while the remaining digits are rotated using a circular shift of one digit to the right.

The pilot-elements matrix *β*_*k*_ corresponding to the matrix *B*_*k*_ is obtained by restricting the values of *w* (and hence the corresponding *z* values) to *w* = 0, 1, …, *p*^*n*−1^ − 1.

Moreover, we note that if we write

62 $$ L_{i} = P^{ - 1} \,G_{i} = P^{n - 1} \,G_{i} $$

and note that *G*_*i*_ is similar in structure to *C*_*N*_, we have

63 $$ z = \left[ {w + \left( {w\,\bmod \,p^{k} } \right)\,\left( {p^{n} - 1} \right)} \right]/p^{k} $$

with *k* = *n* − 1.

To obtain the pilot elements matrix *λ*_*i*_ corresponding to *L*_*i*_ we write

64 $$ z^{\prime } = z\,\bmod \,p^{n - 1} $$

in order to reveal all satellite elements accompanying each pilot element. We then eliminate all the repeated entries in *z*′ and the corresponding *w* values, retaining only pilot elements positions. Alternatively we simply force to zero the digit of weight *n* − 2 in *w* and that of weight *n* − 1 in *z*.

## The CGWP factorization

We presently focus our attention on the matrices

65 $$ B_{k} = C\,H_{k} ;\quad k = 0,1, \ldots ,n - 1. $$

In evaluating the pilot elements coordinates we begin by setting the number of processors *M* = 1. The corresponding *w* − *z* relation of the pilot elements are thus evaluated with *m* = 0. Once this relation has been established it is subsequently used as the reference “*w* − *z* conversion template” to produce the pilot element positions for a general number of *M* = *p*^*m*^ processors. A “right” scan is applied to the matrix in order to produce the *w* − *z* template with an ascending order of *w*. In this scanning type the algorithm advances the first index *w* from zero selecting pilot elements by evaluating their displacement to the right as the second index *z*. Once the template has been evaluated the value *m* corresponding to the number of processors to be dispatched is used to perform successive *p*-ary divisions in proportion to *m* to assign the *M* processors with maximum spacing, leading to maximum possible lengths of memory queues. A “down” scan is subsequently applied, where *p*-ary divisions are applied successively while proceeding downward along the matrix columns, followed by a selection of the desired optimal scan.

The template evaluation and subsequent *p*-ary divisions for the assignment of the *M* processors through a right type scan produce the following hypercube assignments. The assignments are as expected functions of the four variables *n*, *p*, *k* and *m*. The conditions of validity of the different assignments are denoted by numbers and letters for subsequent referencing. With *K* denoting the main clock, the following hypercube transformations are obtained

66 $$ \begin{aligned} K & \simeq \,\left( {j_{n - 1} \ldots j_{m + 1} j_{m} i_{m - 1} \ldots i_{1} i_{0} } \right)_{p} \\ K_{{\overline{n - 1} }} & \simeq \,\left( {0j_{n - 2} \ldots j_{m + 1} j_{m} i_{m - 1} \ldots i_{1} i_{0} } \right)_{p} \\ K_{{\overline{n - 2} \,}} & \simeq \,\left( {j_{n - 1} 0j_{n - 3} \ldots j_{m + 1} j_{m} i_{m - 1} \ldots i_{1} i_{0} } \right)_{p} \\ \end{aligned} $$

1. *k* < *n* − 2
  - *x*: *m* = 0
    67 $$ w \simeq K_{{\overline{n - 1} }} $$
    68 $$ z \simeq \left[ {\left( {I_{{p^{k} }} \times P_{{p^{n - k} }} } \right)K} \right]_{{\overline{n - 2} }} $$
  - *y*: 1 ≤ *m* ≤ *n* – *k* −2
    69 $$ w \simeq \left[ {\left( {P_{{p^{k + 1} }} \times I_{{p^{n - k - 1} }} } \right)\,\prod\limits_{t = 1}^{m - 1} {\left( {I_{{p^{t} }} \times P_{{p^{n - t - 1} }} \times I_{p} } \right)K} } \right]_{{\overline{n - 1} }} $$
    70 $$ z \simeq \left[ {P_{{p^{n} }} \,\prod\limits_{t = 1}^{m - 1} {\left( {I_{{p^{t} }} \times P_{{p^{n - t - 1} }} \, \times \,I_{p} } \right)K} } \right]_{{\overline{n - 2} }} $$
  - $$ z{:}\,n - k - 1 \le m \le n - 1 $$
    71 $$ w \simeq \left[ {\left( {P_{{p^{k + 1} }} \times I_{{p^{n - k - 1} }} } \right)\,\prod\limits_{t = 1}^{m - 1} {\left( {I_{{p^{t} }} \times P_{{p^{n - t - 1} }} \times I_{p} } \right)K} } \right]_{{\overline{n - 1} }} $$
    72 $$ z \simeq \left[ {P_{{p^{n} }} \,\prod\limits_{t = 1}^{m - 1} {\left( {I_{{p^{t} }} \times P_{{p^{n - t - 1} }} \times I_{p} } \right)K} } \right]_{{\overline{n - 2} }} $$
2. $$ k = n - 2 $$
  - $$ u{:}\,m = 0 $$
    73 $$ \begin{aligned} w & \simeq K_{{\overline{n - 1} }} \\ z & \simeq \left[ {\left( {I_{{p^{n - 2} }} \times P_{{p^{2} }} } \right)K} \right]_{{\overline{n - 2} }} \\ \end{aligned} $$
  - $$ v{:}\,m \ge 1 $$
    74 $$ w \simeq \left[ {\,\prod\limits_{t = 0}^{m - 1} {\left( {I_{{p^{t} }} \times P_{{p^{n - t - 1} }} \times I_{p} } \right)K} } \right]_{{\overline{n - 1} }} $$
    75 $$ z \simeq \left[ {P_{{p^{n} }} \,\prod\limits_{t = 1}^{m - 1} {\left( {I_{{p^{t} }} \times P_{{p^{n - t - 1} }} \times I_{p} } \right)K} } \right]_{{\overline{n - 2} }} $$
    $$ t{:}\,k = n - 1 $$
    76 $$ w = z\, \simeq \,\left[ {\,\prod\limits_{t = 0}^{m - 1} {\left( {I_{{p^{t} }} \times P_{{p^{n - t - 1} }} \times I_{p} } \right)K} } \right]_{{\overline{n - 1} }} $$

Evaluated, these hypercubes yield the following pilot elements assignments:

- $$ x{:}\,\left( {k < n - 2\,,\quad m = 0} \right) $$
  77 $$ w = \sum\limits_{j = 0}^{n - 2} {p^{t} \,j_{t} } $$
  78 $$ z = \sum\limits_{j = 0}^{k - 1} {p^{t} \,j_{t} + p^{n - 1} \,j_{k} } + \sum\limits_{t = k + 1}^{n - 2} {p^{t - 1} \,j_{t} } $$
- $$ y{:}\,k < n - 2\,,\quad 1 \le m \le n - k - 2 $$
  79 $$ w = p^{k} \,i_{0} + \sum\limits_{s = 1}^{m - 1} {p^{n - 1 - s} \,i_{s} \, + \,\sum\limits_{t = m}^{m + k - 1} {p^{t - m} \,j_{t} } } \, + \sum\limits_{t = m + k}^{n - 2} {p^{t - m + 1} \,j_{t} } $$
  80 $$ z = p^{n - 1} \,i_{0} + \sum\limits_{s = 1}^{m - 1} {p^{n - 2 - s} } \,i_{s} + \sum\limits_{t = m}^{n - 2} {p^{t - m} \,j_{t} } $$
- $$ z{:}\,k < n - 2\,,\quad n - k - 1 \le m \le n - 1 $$
  81 $$ w = p^{k} \,i_{0} + \sum\limits_{s = 1}^{n - k - 2} {p^{n - 1 - s} } \,i_{s} + \sum\limits_{s = n - k - 1}^{m - 1} {p^{n - 2 - s} } \,i_{s} + \sum\limits_{s = m}^{n - 2} {p^{t - m} } \,j_{t} $$
  82 $$ z = p^{n - 1} \,i_{0} + \sum\limits_{s = 1}^{m - 1} {p^{n - 2 - s} } \,i_{s} + \sum\limits_{t = m}^{n - 2} {p^{t - m} } \,j_{t} $$
- $$ u{:}\,k = n - 2\,,\quad m = 0 $$
  83 $$ w = \sum\limits_{t = 0}^{n - 2} {p^{t} j_{t} } $$
  84 $$ z = \sum\limits_{j = 0}^{n - 3} {p^{t} j_{t} } + p^{n - 1} \,j_{n - 2} $$
- $$ v{:}\,k = n - 2\,,\quad m \ge 1 $$
  85 $$ w = \sum\limits_{s = 0}^{m - 1} {p^{k - s} \,i_{s} } + \sum\limits_{t = m}^{n - 2} {p^{t - m} \,j_{t} } $$
  86 $$ z = p^{n - 1} \,i_{0} + \sum\limits_{s = 1}^{m - 1} {p^{k - s} \,i_{s} } + \sum\limits_{t = m}^{n - 2} {p^{t - m} \,j_{t} } $$
- $$ t{:}\,k = n - 1 $$
  87 $$ w = z = \sum\limits_{s = 0}^{m - 1} {p^{n - 2 - s} \,i_{s} } + \sum\limits_{t = m}^{n - 2} {p^{t - m} \,j_{t} }. $$

## Row and column scans for optimal assignment

A processor is considered optimal if it requires a minimum of memory partitions, is shuffle free, meaning the absence of clock times used uniquely for shuffling, and produces an ordered output given an ordered input. It is shown in Corinthios (1994) that *p*^2^-optimal algorithms and processors lead to a minimum number of *p*^2^ partitions of *N*/*p*^2^ queue length each. With *M* = *p*^*m*^ base-*p* processors operating in parallel the number of partitions increases to *p*^*m*+2^ and the queue length of each partition reduces to *N*/*p*^*m*+2^.

An optimal multiprocessing algorithm should satisfy such optimality constraints. The horizontal spacing between simultaneously accessed pilot elements defines the input memory queue length. The vertical spacing defines the output memory queue length. With *M* processors applied in parallel the horizontal spacing between the accessed elements will be referred to as the “input pitch”, while the vertical spacing as the “output pitch”.

By choosing the pilot elements leading to the maximum possible pitch, which is the highest of the two values: the minimum input pitch and minimum output pitch, optimality in the form of *N*/*p*^*m*+2^ queue length is achieved.

We note that optimal minimum memory queue length MMQL satisfies

$$ MMQL = \left\{ {\begin{array}{*{20}l} {p^{n - m - 2} ,} \hfill & {\quad m \le n - 2} \hfill \\ {1,} \hfill & {\quad m = n - 1} \hfill \\ \end{array} } \right. $$

The following algorithm, Algorithm 2, describes this approach to state assignment optimality.
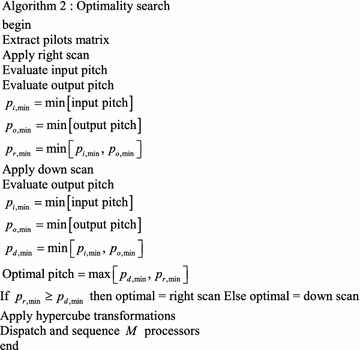


In following the algorithm we note that in the validity condition y of the *B*_*k*_ matrix *y* : 1 ≤ *m* ≤ *n* − *k* − 2 the results obtained are such that the digit *i*_0_ of *w* is of a weight *p*^*k*^. Hence the input pitch is *p*^*k*^ while the output pitch which can be deduced from the position of *i*_0_ in *z* is *p*^*n*−1^, that is, maximal possible. The input pitch is thus function of *k* and can be low if *k* is small. By performing a down scan of *B*_*k*_ we obtain the following solution:

$$ \begin{aligned} & k < n - 2 \\ & y{:}\,1 \le m \le n - k - 2 \\ & \quad w{:}\,0\quad \quad i_{0} \quad \quad i_{1} \quad \quad \ldots \quad \quad i_{m - 1} \quad \quad j_{n - 2} \quad \quad \ldots \\ & \quad \quad j_{m + 1} \quad j_{m} \\ & \quad z{:}\,j_{m + k} \quad 0\quad \quad i_{0} \quad \quad i_{1} \quad \quad \ldots \quad \quad i_{m - 1} \quad \quad j_{n - 2} \quad \quad \ldots \quad \quad j_{m + k + 1} \quad j_{m + k - 1} \quad \ldots \\ & \quad \quad j_{m + 1} \quad j_{m} \\ \end{aligned} $$

where now it is *i*_*m*−1_ that leads to a minimum pitch and it has a weight of *p*^*n*−*m*−1^ in *w* and *p*^*n*−*m*−2^ in *z*. We deduce that the minimum pitch in this solution is *p*^*n*−*m*−2^, which is the optimal sought. The same reasoning leads to the optimal assignment for the case

$$ k < n - 2 $$

$$ \begin{aligned} & z{:}\,n - k - 1 \le m \le n - 1 \\ & w{:}\,0\quad \quad i_{0} \quad \quad i_{1} \quad \quad \ldots \quad \quad \ldots \quad \quad i_{m - 1} \quad \quad j_{n - 2} \quad \quad \ldots \\ & \quad j_{m + 1} \quad j_{m} \\ & z{:}\,i_{n - 2 - k} \quad 0\quad \quad i_{0} \quad \quad i_{1} \quad \quad \ldots \quad \quad i_{n - 3 - k} \quad \quad i_{n - 1 - k} \quad \quad i_{n - k} \quad \quad \ldots \quad \quad i_{m - 1} \quad \quad j_{n - 2} \quad \quad \ldots \\ & \quad \quad j_{m + 1} \quad j_{m} \\ \end{aligned} $$

These are the only two cases of the matrix that need be thus modified for optimality. All results obtained above for the other validity conditions can be verified to be optimal.

### Matrix span

In the above from one iteration to the next the value of *k* is incremented. In each iteration once the pilot element matrix coordinates (*w*, *z*) are determined as shown above each processor accesses *p* elements spaced by the row span starting with the pilot element and writes its *p* outputs at addresses spaced by the column span. The row and column spans of a matrix are evaluated as is shown in Corinthios (1994). In particular we note that the matrix

88 $$ B_{k} = CH_{k} $$

has the same column span as that of *C*, namely *σ*_*c*_(*B*_*k*_) = *σ*_*c*_(*C*) = *p*^*n*−1^. The row span of *B*_*k*_ is evaluated by noticing that *B*_*k*_ has the same structure as *C* with its columns permuted in accordance with the order implied by

89 $$ H_{k}^{ - 1} = I_{{p^{k} }} \times P_{{p^{n - k} }}^{ - 1} $$

The transformation of the hypercube (*i*_*n*−1_…*i*_1_*i*_0_) corresponding to *H*_*k*_^−1^ is one leading to a most significant digit equal to *i*_*n*−2_. Since this digit changes value from 0 to 1 in a cycle length of *p*^*n*−2^ we deduce that the row span of all the *B*_*k*_ matrices is simply

90 $$ \sigma_{R} \left( {B_{k} } \right) = p^{n - 2} . $$

Each processing element thus accesses *p* operands spaced *p*^*n*−2^ points apart and writes their *p* outputs at points which are *p*^*n*−1^ points apart.

## The CGWK factorization

The sampling matrices of the CGWK factorization are more complex in structure than the other generalised spectral analysis matrices. They are defined by

91 $$ \Gamma _{i} = P^{ - 1} G_{i} S_{i + 1} $$

Let

92 $$ L_{i} \, \triangleq \,P^{ - 1} \,G_{i} $$

we have

93 $$ \Gamma _{i} = L_{i} \,S_{i + 1} . $$

We note that the sampling matrix *G*_*i*_ has the same structure in poles and zeros, that is, in the positions of non-zero and zero elements respectively, as that of the matrix *C*_*N*_. We can write for the matrix *G*_*i*_

94 $$ \begin{aligned} w_{{G_{i} }} & \simeq \left( {j_{n - 2} \, \ldots j_{1} \,j_{0} } \right) \\ z_{{G_{i} }} & \simeq \left( {j_{n - 2} \ldots j_{1} \,j_{0} } \right) \\ \end{aligned} $$

as the pilot elements positions.

Given the definition of the matrix *L*_*i*_ a hypercube rotation corresponding to the matrix *P*^−1^ would yield the *w* and *z* values of *L*_*i*_ as:

95 $$ \begin{aligned} w_{{L_{i} }} & \simeq \left( {j_{n - 2} \,0\,j_{n - 3} \, \ldots j_{1} \,j_{0} } \right) \\ z_{{L_{i} }} & = P^{ - 1} \,w_{{L_{i} }} \simeq \left( {0\,j_{n - 3} \ldots j_{1} \,j_{0} \,j_{n - 2} } \right) \\ \end{aligned} $$

Alternatively, a z-ordered counterpart can be written as:

96 $$ \begin{aligned} z_{{L_{i} }} & \simeq \left( {0\,j_{n - 2} \ldots j_{i} \,j_{0} } \right) \\ w_{{L_{i} }} & \simeq \left( {j_{0} \,0\,j_{n - 2} \ldots j_{2} \,j_{1} } \right) \\ \end{aligned} $$

Similarly, the matrix Γ_0_ = *G*_0_*S*_1_ which is obtained from *G*_0_ by permuting its columns according to the order dictated by

97 $$ S_{1}^{ - 1} = P_{{p^{n - 1} }}^{ - 1} \times I_{p} $$

leads to the *m* = 0 template assignment

98 $$ w_{{\Gamma _{0} }} \simeq \left( {0\,j_{n - 2} \ldots j_{1} \,j_{0} } \right) $$

99 $$ z_{{\Gamma _{0} }} \, = S_{1} \,w_{{\Gamma _{0} }} \simeq \left( {0\,j_{0} \,j_{n - 2} \ldots j_{2} \,j_{1} } \right) $$

and a similar z-ordered state assignment counter part.

For

100 $$ \Gamma _{k} = G_{0} S_{k} \,,\quad k\text{ > }0 $$

we have

101 $$ S_{k}^{ - 1} = I_{{p^{k - 1} }} \times P_{{p^{n - k} }}^{ - 1} \times I_{p} $$

which leads to the state template assignment

102 $$ \begin{aligned} w_{{\Gamma _{k} }} & \simeq w_{{L_{i} }} \simeq \left( {j_{n - 2} \,0\,j_{n - 3} \ldots j_{1} \,j_{0} } \right)\,, \\ z_{{\Gamma _{k} }} & = S_{k + 1} \,z_{{L_{i} }} \simeq \left( {0\,j_{k - 1} \,j_{n - 3} \, \ldots j_{k + 1} \,j_{k} \,j_{k - 2} \ldots j_{1} \,j_{0} \,j_{n - 2} } \right);\quad k\text{ > }0 \\ \end{aligned} $$

With *m* made variable a right scan yields the following expressions for the different validity conditions.

### The $\Gamma _{k}$ transformations

1. $$ k = 0 $$
  $$ a{:}\,k = 0,m = 0 $$
    103 $$ \begin{aligned} w\, &\simeq \,K_{{\overline{n - 1} }} \hfill \\ z\, &\simeq P_{{p^{n} }} \,K_{{\overline{n - 1} }} \equiv \left[ {\left( {P_{{p^{n - 1} }} \, \times I_{p} } \right)K} \right]_{{\overline{n - 1} }} \hfill \\ \end{aligned} $$
  $$ b{:}\,k = 0,m \ge 2 $$
    104 $$ w \simeq \,\left[ {\prod\limits_{t = 1}^{m - 1} {\left( {I_{{p^{t} }} \times P_{{p^{n - t - 1} }} \, \times \,I_{p} } \right)} \,K} \right]_{{\overline{n - 1} }} $$
    105 $$ z\, \simeq \,\left[ {\prod\limits_{t = 0}^{m - 1} {\left( {I_{{p^{t} }} \times P_{{p^{n - t - 1} }} \times I_{p} } \right)} \,K} \right]_{{\overline{n - 1} }} $$
2. $$ 1 \le k \le n - 3 $$
  $$ c{:}\,m = 0 $$
    106 $$ w\, \simeq \,\left[ {\left( {I_{{p^{n - 2} }} \times P_{{p^{2} }} } \right)K} \right]_{{\overline{n - 2} }} $$
    107 $$ z\, \simeq \,\left[ {\left( {I_{{p^{k} }} \times P_{{p^{n - k - 1} }} \times I_{p} } \right)\,\left( {P_{{p^{n - 1} }}^{ - 1} \times I_{p} } \right)K} \right]_{{\overline{n - 1} }} $$
  $$ d{:}\,m = 1 $$
    108 $$ w\, \simeq \,\left[ {\left( {I_{{p^{n - 2} }} \times P_{{p^{2} }} } \right)\,\left( {P_{{p^{k} }} \times I_{{p^{n - k} }} } \right)K} \right]_{{\overline{n - 2} }} $$
    109 $$ z\, \simeq \,\left[ {\left( {I_{p} \times P_{{p^{n - 2} }} \times I_{p} } \right)\,\left( {P_{{p^{n - 1} }}^{ - 1} \times I_{p} } \right)K} \right]_{{\overline{n - 1} }} $$
  $$ e{:}\,m \ge 2 $$
    110 $$ z\, \simeq \,\left[ {\left( {P_{{p^{n - 1} }} \times I_{p} } \right)\,\prod\limits_{t = 2}^{m - 1} {\left( {I_{{p^{t} }} \times P_{{p^{n - t - 1} }} \times I_{p} } \right)} \,K} \right]_{{\overline{n - 1} }} $$
  - $$ m \ge n - k $$
    111 $$ w \simeq \left[ {\left( {P_{{p^{k} }} \times I_{{p^{n - k} }} } \right)\prod\limits_{t = 1}^{m - 1} {\left( {I_{{p^{t} }} \times P_{{p^{n - t - 1} }} \times I_{p} } \right)K} } \right]_{{\overline{n - 2} }} $$
  - $$ 2 \le m \le n - k $$
    112 $$ w\, \simeq \,\left[ {\left( {P_{{p^{k} }} \times I_{{p^{n - k} }} } \right)\prod\limits_{t = 1}^{m - 1} {\left( {I_{{p^{t} }} \times P_{{p^{n - t - 1} }} \times I_{p} } \right)} \,K} \right]_{{\overline{n - 2} }} $$
3. $$ k \ge n - 2 $$
  113 $$ w\, \simeq \,\left[ {\left( {I_{{p^{n - 2} }} \times P_{{p^{2} }} } \right)K} \right]_{{\overline{n - 2} }} $$
  114 $$ z\, \simeq \,\left[ {\left( {P_{{p^{n - 1} }}^{ - 1} \times I_{p} } \right)\,K} \right]_{{\overline{n - 1} }} $$
  $$ g{:}\,m = 1 $$
  115 $$ w\, \simeq \left[ {\left( {I_{{p^{2} }} \times P_{{p^{n - 2} }} } \right)\,\left( {P_{{p^{n - 2} }} \times I_{{p^{2} }} } \right)K} \right]_{{\overline{n - 2} }} $$
  116 $$ z\, \simeq \,\left[ {\left( {P_{{p^{n - 2} }}^{ - 1} \times I_{{p^{2} }} } \right)\,\left( {P_{{p^{n - 1} }} \times I_{p} } \right)K} \right]_{{\overline{n - 1} }} $$
  $$ h{:}\,m \ge 2 $$
  117 $$ w\, \simeq \,\left[ {\left( {P_{{p^{n - 2} }} \times I_{{p^{2} }} } \right)\prod\limits_{t = 1}^{m - 1} {\left( {I_{{p^{t} }} \times P_{{p^{n - t - 1} }} \times I_{p} } \right)} \,K} \right]_{{\overline{n - 2} }} $$
  $$ i{:}\,2 \le m \le n - 2 $$
  118 $$ z \simeq \,\left[ {\left( {P_{{p^{n - 1} }} \times I_{p} } \right)\prod\limits_{t = 2}^{m - 1} {\left( {I_{{p^{t} }} \times P_{{p^{n - t - 1} }} \times I_{p} } \right)} \,K} \right]_{{\overline{n - 1} }} $$
  $$ j{:}\,m = n - 1 $$
  119 $$ z\, \simeq \,\left[ {\left( {P_{{p^{n - 1} }} \times I_{p} } \right)\prod\limits_{t = 2}^{m - 1} {\left( {I_{{p^{t} }} \times P_{{p^{n - t - 1} }} \times I_{p} } \right)} \,K} \right]_{{\overline{n - 1} }} $$

### CGWK optimal assignments

A “down” scan of the Γ_*k*_ matrix yields optimal assignments for two validity conditions:

1. $$ k = 0 $$
  $$ a{:}\,k = 0\,,\quad m = 1 $$
    $$ \begin{aligned} & w{:}\,0\quad \quad i_{0} \quad \quad j_{n - 2} \quad \quad \ldots \quad \quad j_{2} \quad \quad j_{1} \\ & z{:}\,0\quad \quad j_{1} \quad \quad i_{0} \quad \quad j_{n - 2} \quad \quad \ldots \quad \quad j_{3} \quad \quad j_{2} \\ \end{aligned} $$
  $$ b{:}\,k = 0\,,\quad m \ge 2 $$
    $$ \begin{aligned} & w{:}\,0\quad \quad i_{0} \quad \quad i_{1} \quad \quad \ldots \quad \quad i_{m - 1} \quad \quad j_{n - 2} \quad \quad \ldots \quad \quad j_{m + 1} \quad \quad j_{m} \\ & z{:}\,0\quad \quad j_{m} \quad \quad i_{0} \quad \quad i_{1} \quad \quad \ldots \quad \quad i_{m - 2} \quad \quad j_{n - 2} \quad \quad \ldots \quad \quad j_{m + 1} \\ \end{aligned} $$

All other assignments generated by the “right” scan are optimal and need not be replaced.

### The CGWK matrix spans

Using the same approach we deduce the spans of the different CGWK factorization matrices. We have

120 $$ \sigma_{R} \left( {L_{i} } \right) = \sigma_{R} \left( {G_{i} } \right) = p^{n - 1} $$

121 $$ \sigma_{c} \left( {L_{i} } \right) = p^{n - 2} $$

122 $$ \sigma_{R} \left( {\Gamma _{0} } \right) = p^{n - 1} $$

123 $$ \sigma_{c} \left( {\Gamma _{0} } \right) = \sigma_{c} \left( {G_{0} } \right) = p^{n - 1} $$

and

124 $$ \sigma_{R} \left( {\Gamma _{i} } \right) = p^{n - 1} $$

125 $$ \sigma_{c} \left( {\Gamma _{i} } \right) = \sigma_{c} \left( {P^{ - 1} G_{i} } \right) = \sigma_{c} \left( {L_{i} } \right) = p^{n - 2} . $$

## FPGA configuration

Configuring FPGAs to execute digital signal processing algorithms in real time has been rendered readily accessible through model simulation using Matalb^©^ and Simulink. In what follows we summarize results obtained in configuring Xilinx FPGA boards and particular the Artix-7 Nexys 4 DDR platform. In these applications the basic Discrete Chrestenson transform matrices with *M* = 1, *p* = 2 and *n* = 5 defining 32-point transforms both as the Discrete Fourier transforms and Walsh–Hadamard transforms are presented. In both cases the transform of ramp is evaluated.

**Fig. 1:**
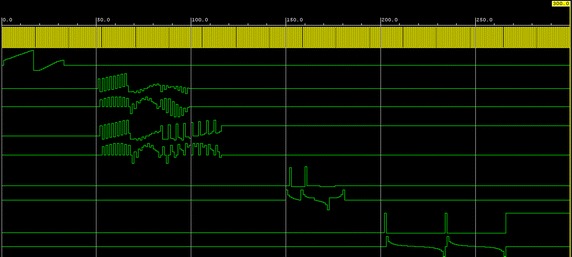
Waveforms of successive iterations and the final result in the case of the evaluation of the CGW discrete Fourier transform

Figure 1 shows the waveforms which appear in the successive iterations and the final result in the case of the evaluation of the CGW Discrete Fourier transform.

**Fig. 2:**
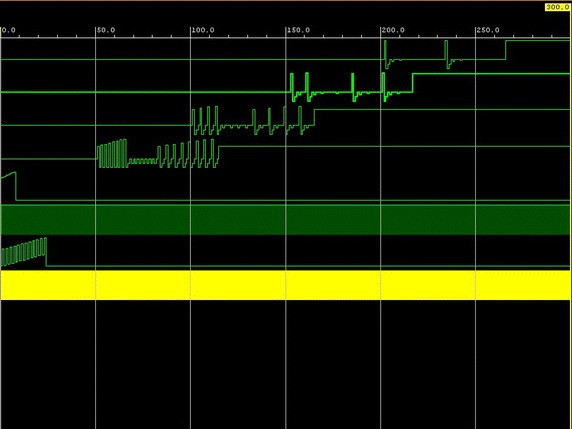
Waveforms of successive iterations and the final result in the case of the evaluation of the CGWP Walsh–Hadamard transform

The corresponding waveforms in the case of the CGWP Walsh–Hadamard transform successive iterations and the final result are shown in Fig. 2.

## Conclusion

A formalism and an algorithm for the parallel implementation of the Chrestenson transform employing rotations of a general-base hypercube and their embedding into FPGA architectures has been presented. Closed-form general-radix factorizations of the transformation matrices, showing processor architecture and sequencing of an arbitrary number *M* = *p*^*n*−1^ of general-base processors have been obtained. Pilot elements addresses and matrix spans to locate their satellites are automatically generated for dispatching and sequencing the parallel processors.
